# Supplementary figures and images for: Mathematical model reveals that heterogeneity in the number of ion transporters regulates the fraction of mouse sperm capacitation
Source: PLoS One. 2021 Nov 18;16(11):e0245816. doi: 10.1371/journal.pone.0245816 (PMC8601445; doi:10.1371/journal.pone.0245816)

ChAcc

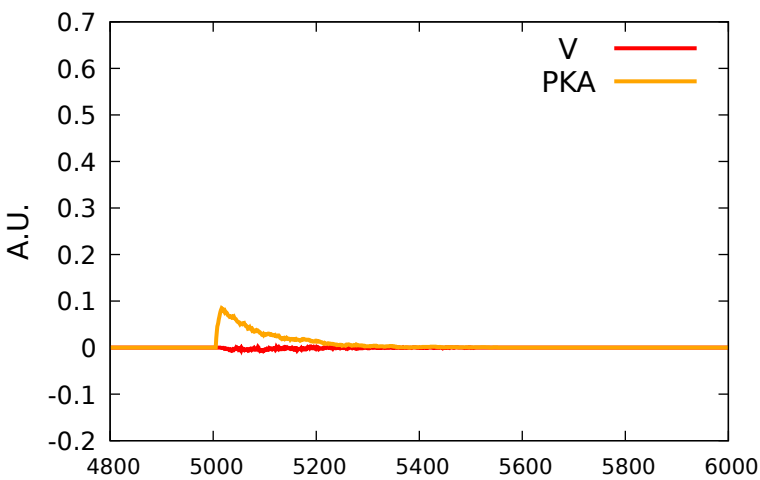 $\text{HCO}_3\text{e}$ 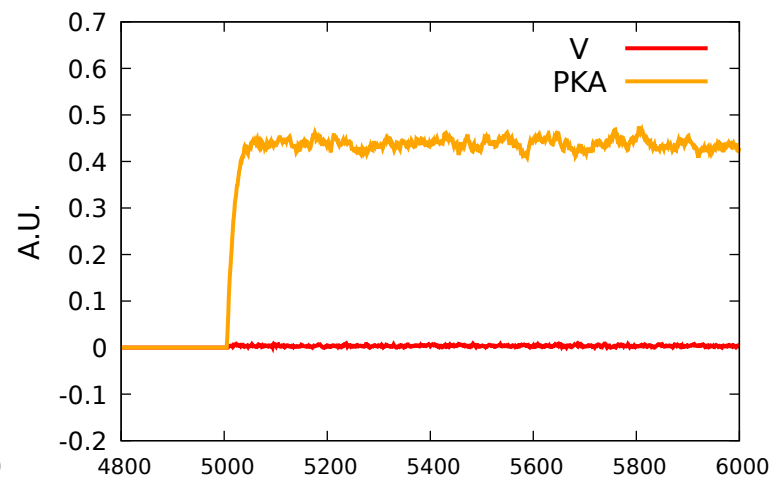ChAcc+ $\text{HCO}_3\text{e}$ 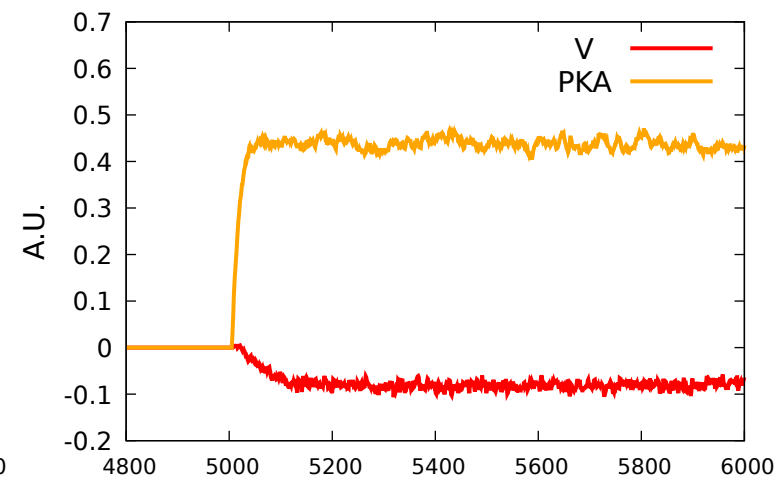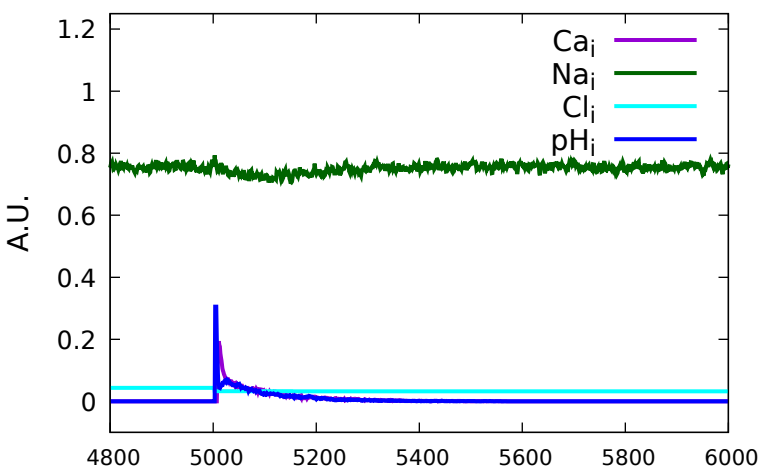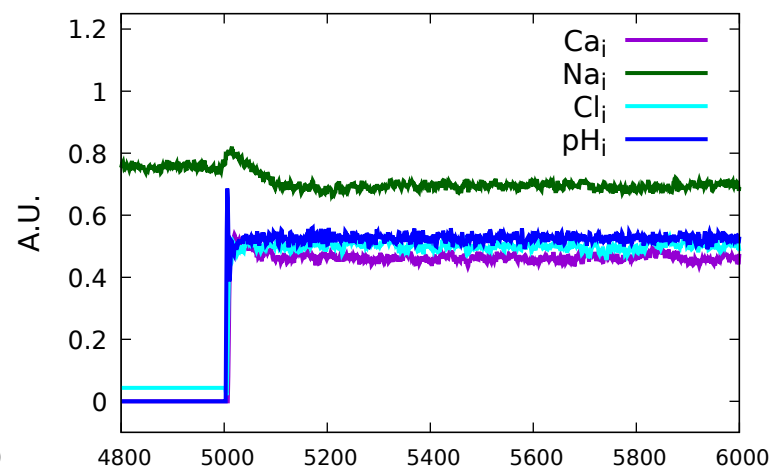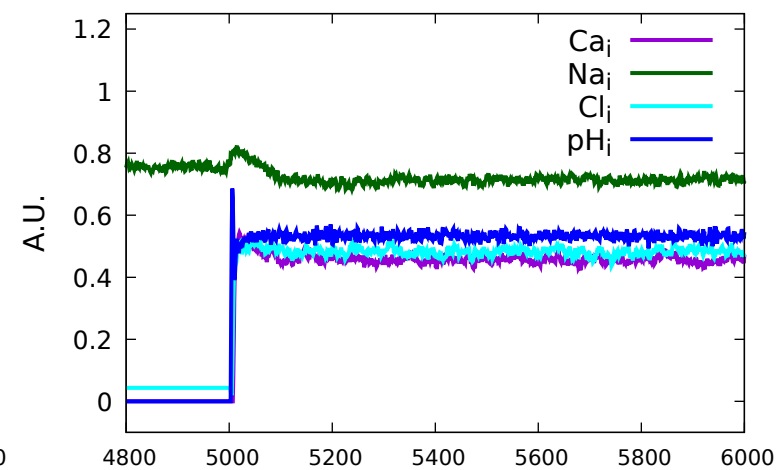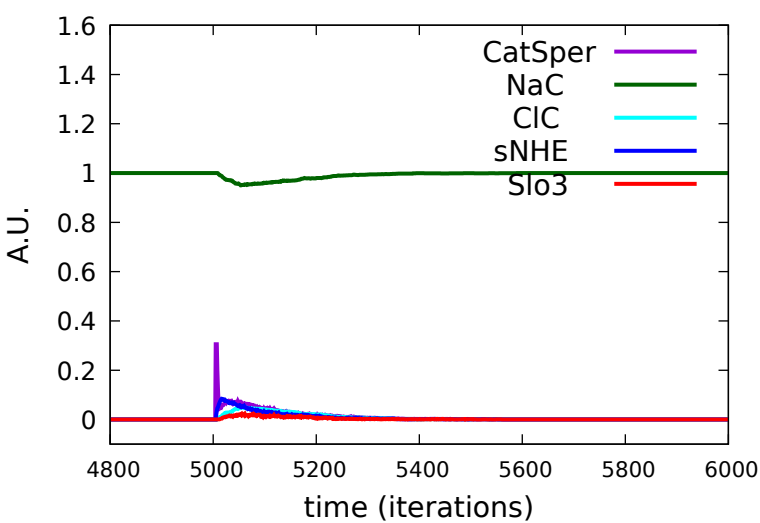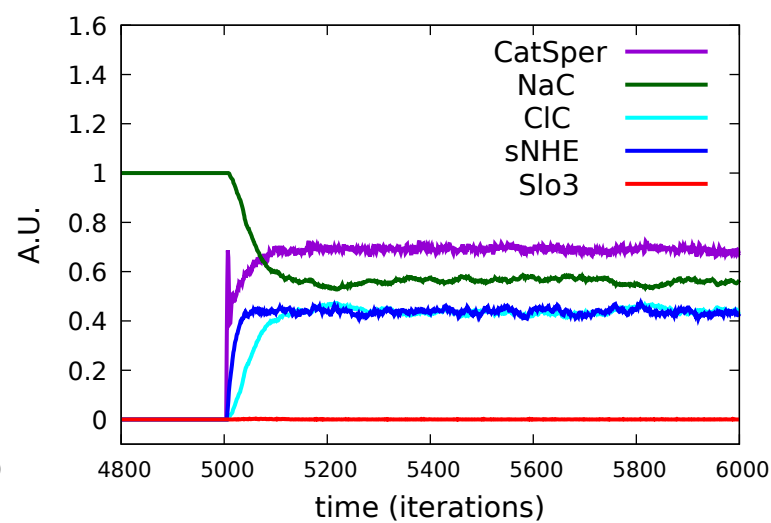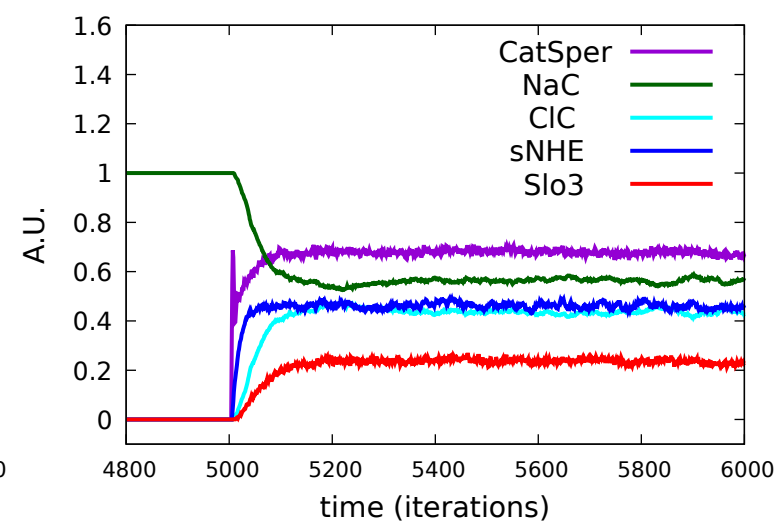

Supplement: S1 Fig — Averaged time series of a select set of variables from a WT sperm population, without variability in their ion transporter weights, subject to external stimulation. In the simulations, stimuli are introduced at time t = 5 × 10^3^ and consist of cholesterol acceptor-only (first column), bicarbonate-only (second column), or both (third column). The qualitative trends of each variable were used to validate our model and are summarized in Table 1 of Section 2.2.1. Population size is 2 × 10^3^ sperm. (PDF) [file pone.0245816.s002.pdf]

ChAcc

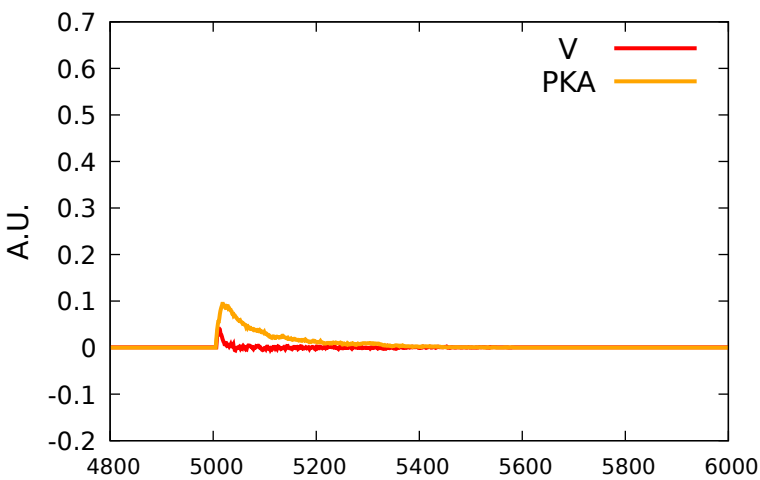 $\text{HCO}_3\text{e}$ 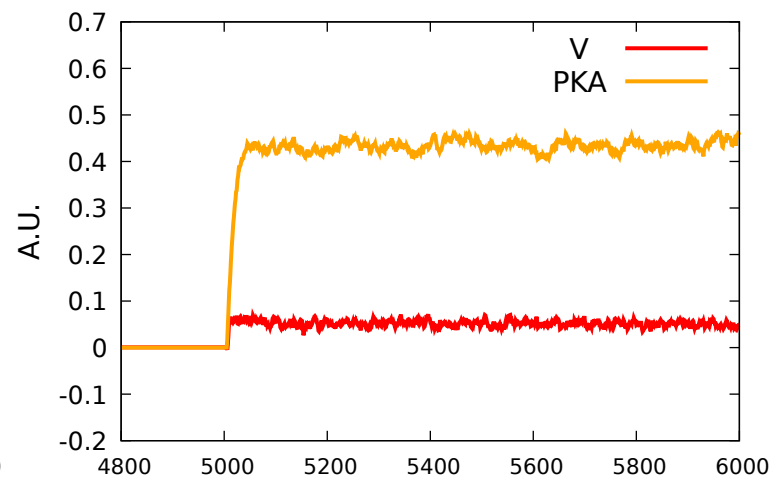ChAcc+ $\text{HCO}_3\text{e}$ 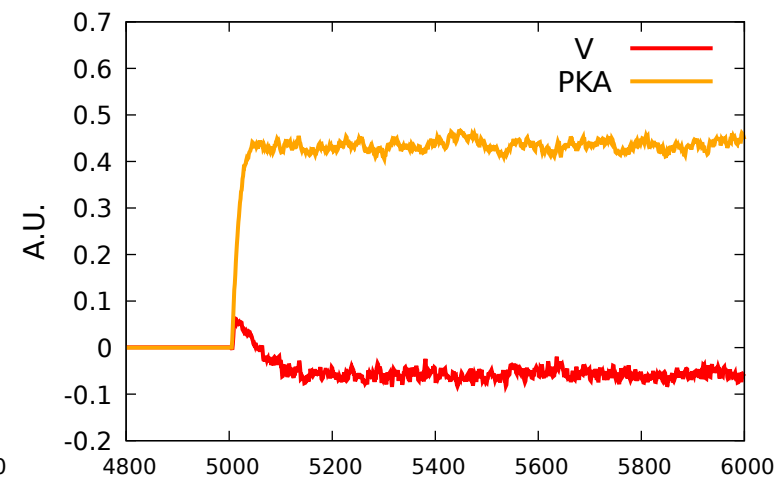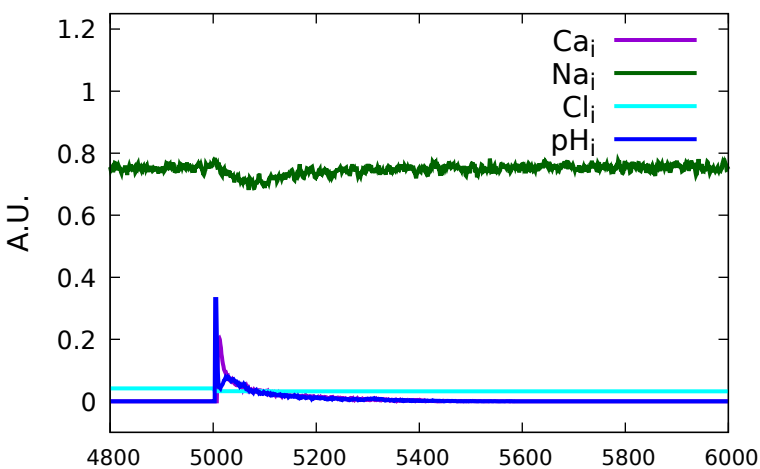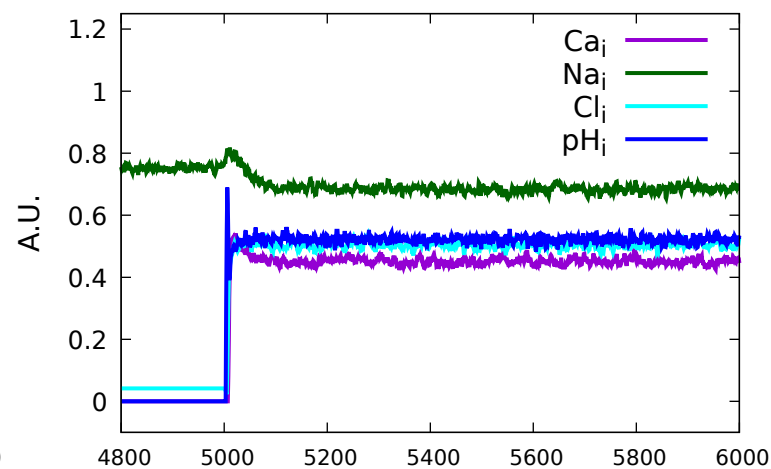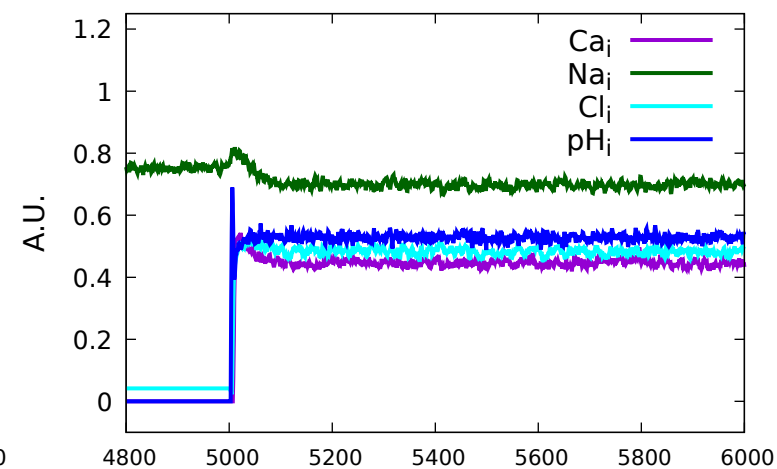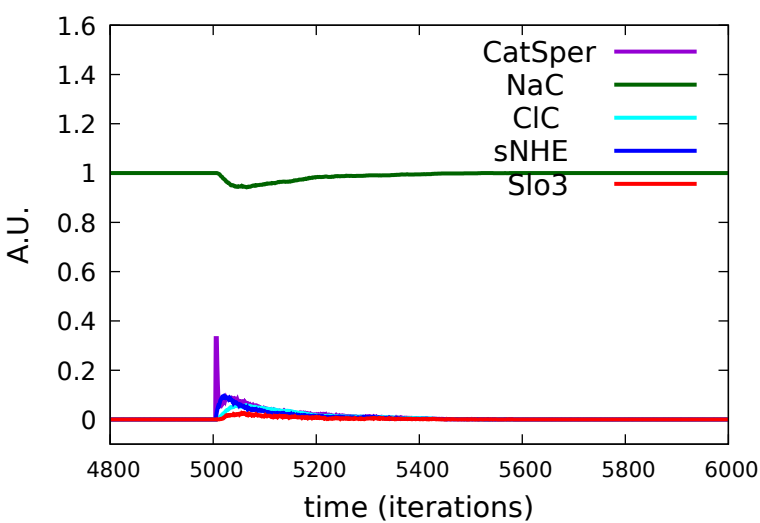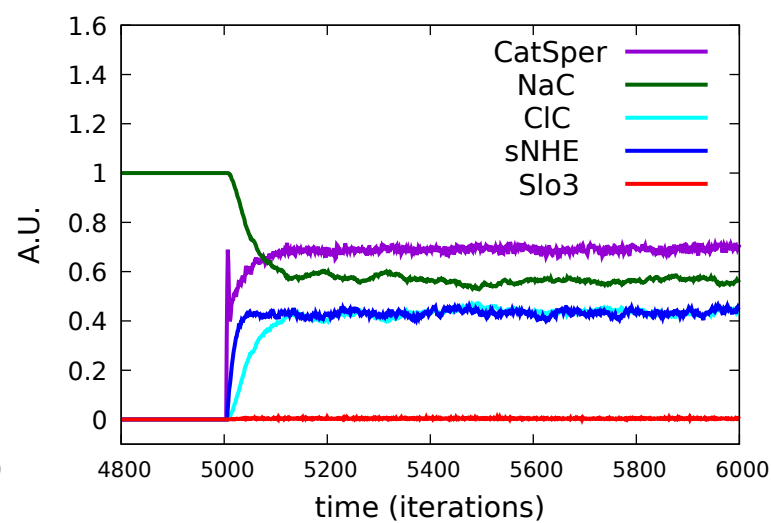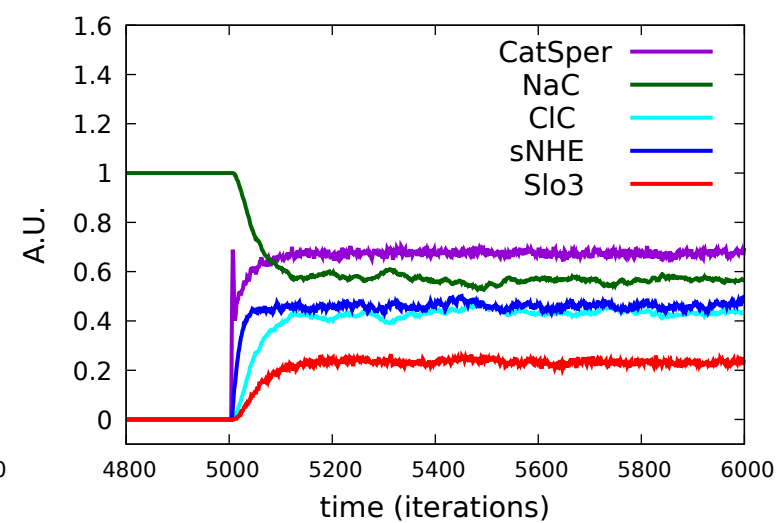

Supplement: S2 Fig — Averaged time series of a select set of variables from a WT sperm population, with variability in ion transport weights sampled from a Gaussian distribution with standard deviation D = 0.25, under several stimuli. Simulations shown in the panels are performed as in S1 Fig. Notice the similarity of both figures, trends are preserved under the inclusion of the above mentioned variability. (PDF) [file pone.0245816.s003.pdf]

WT

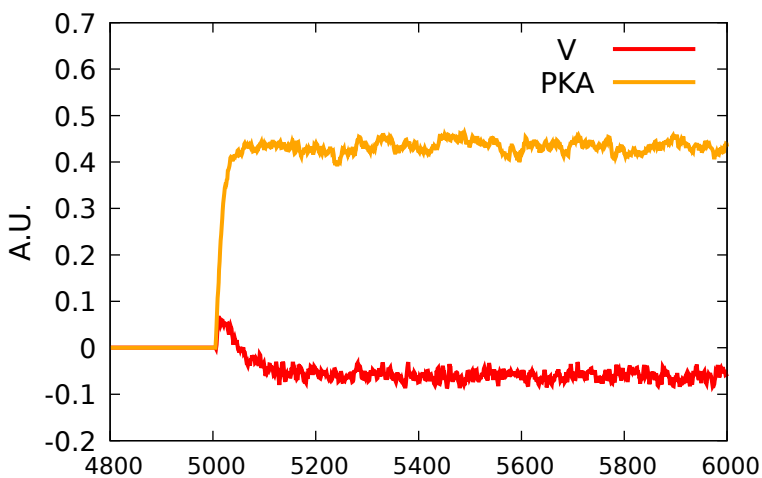CatSper<sup>LOF</sup>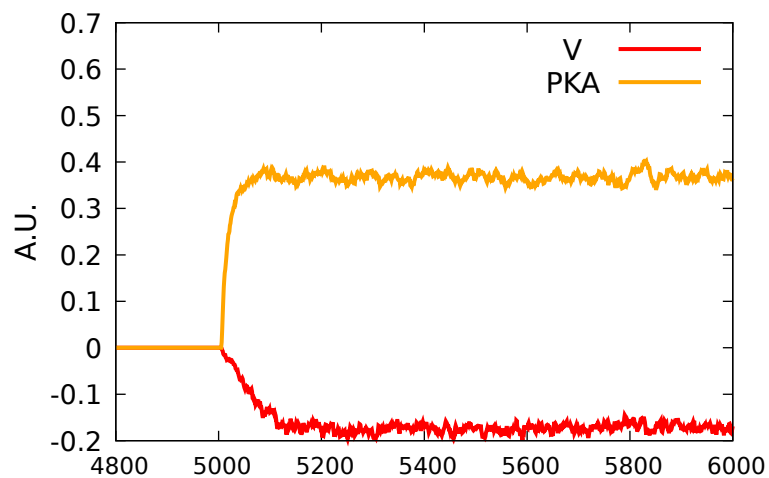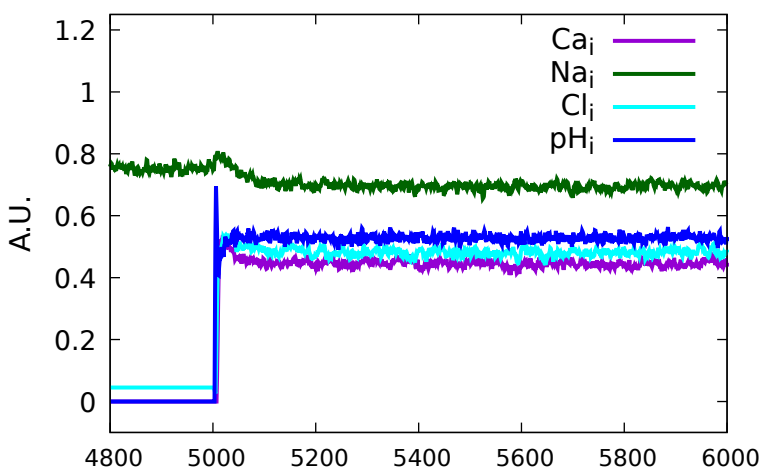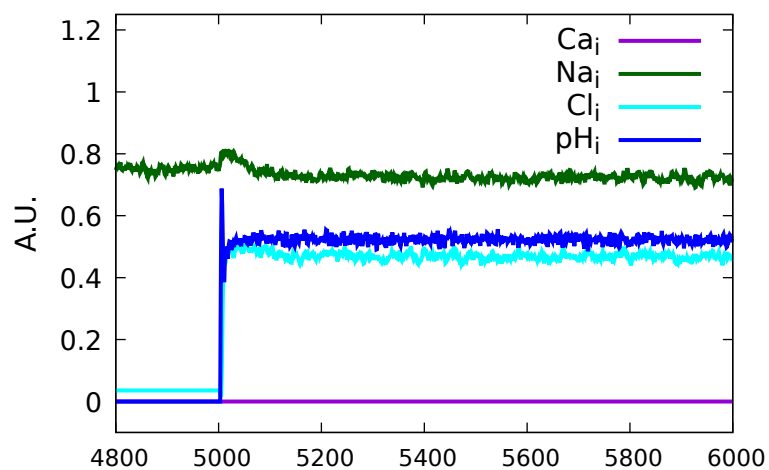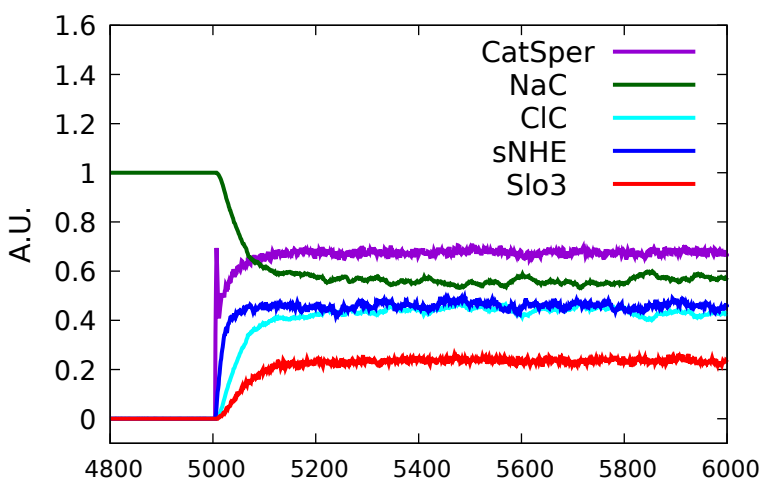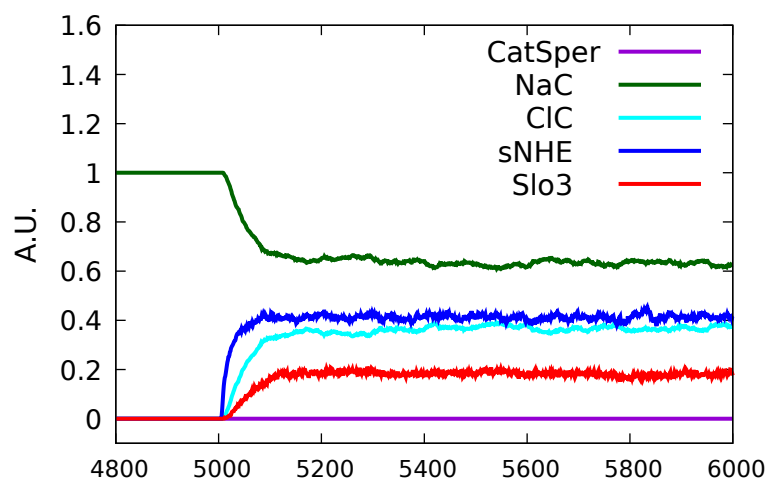

Supplement: S3 Fig — Averaged time series with variability, under an external stimulation of the addition of cholesterol acceptors and higher bicarbonate levels in the extracellular medium, of a select set of variables in a WT network as reference (left column), and in a CatSperLOF network variant (right column). Under CatSperLOF, membrane potential V hyperpolarizes, Cai goes to basal levels, Nai decreases, whereas Cli, pHi and PKA activity increase. Population size is 2 × 10^3^ sperm, the standard deviation used in introducing variability on ion transporter weights is D = 0.25. (PDF) [file pone.0245816.s004.pdf]

WT

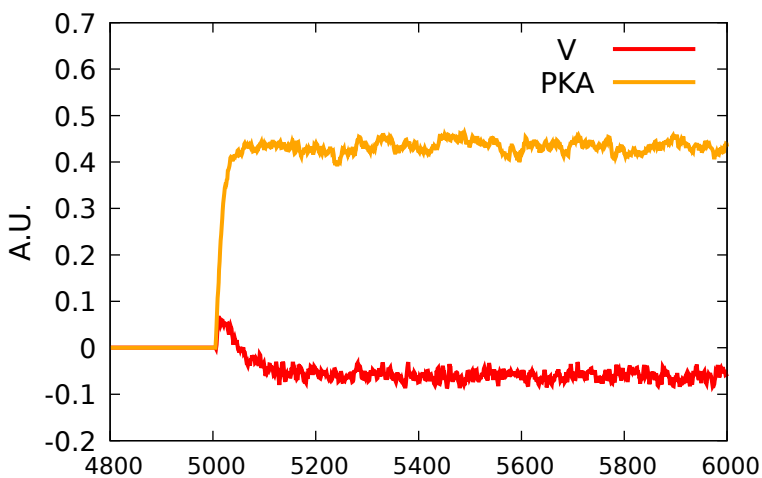CIC<sup>LOF</sup>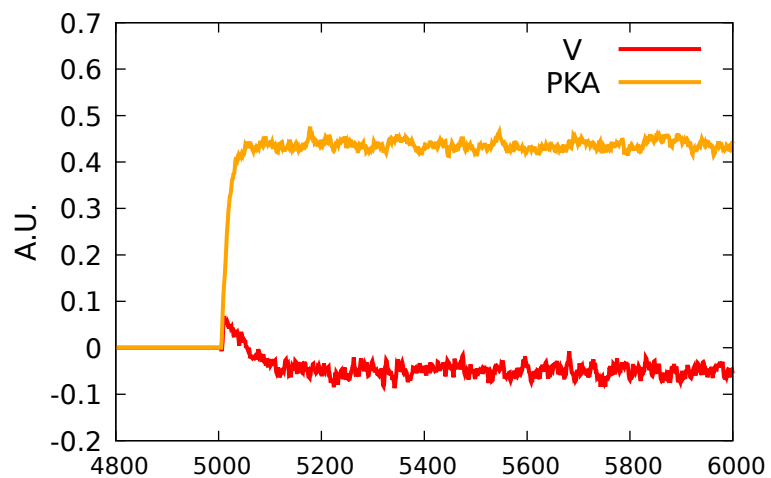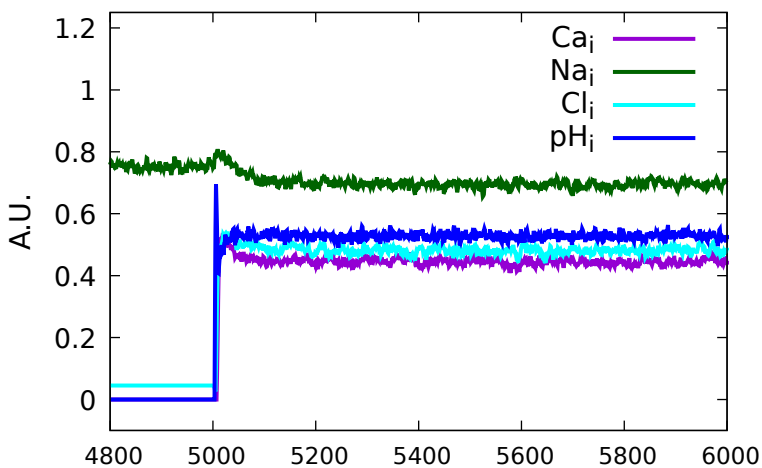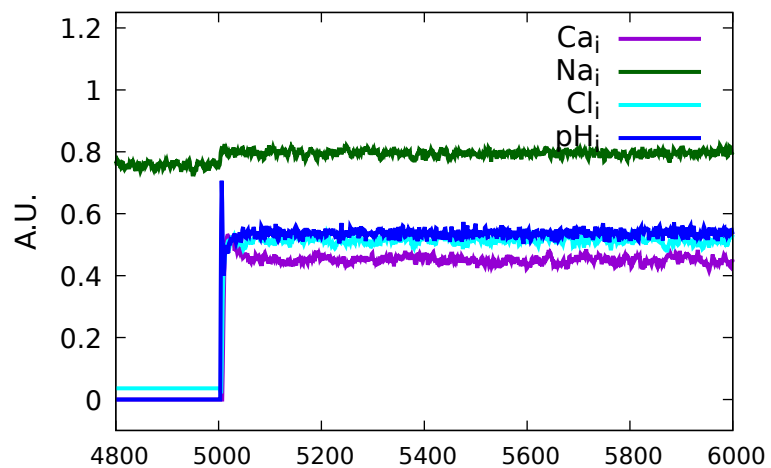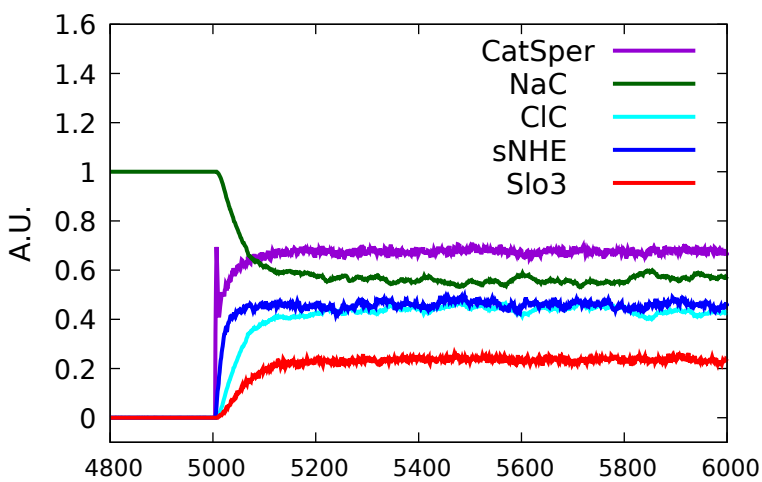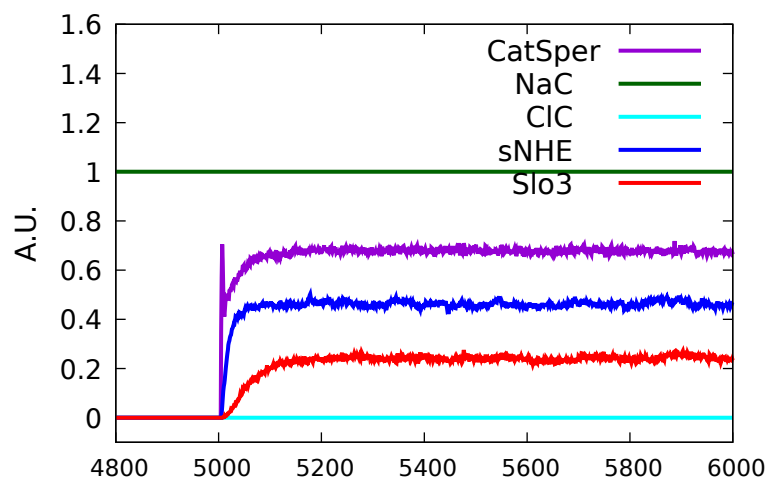

Supplement: S4 Fig — Comparison of the averaged time series with variability, under an external stimulation of the addition of cholesterol acceptors and higher bicarbonate levels in the extracellular medium, of select nodes under WT conditions (first column) and loss of function (LOF) of ClC (second column). Notice that under LOF membrane potential V hyperpolarizes, Nai goes to basal levels, Cai, Cli, pHi and PKA activity increase. Population size is 2 × 10^3^ sperm, the standard deviation used for variability on ion transporter weights is D = 0.25. (PDF) [file pone.0245816.s005.pdf]

WT

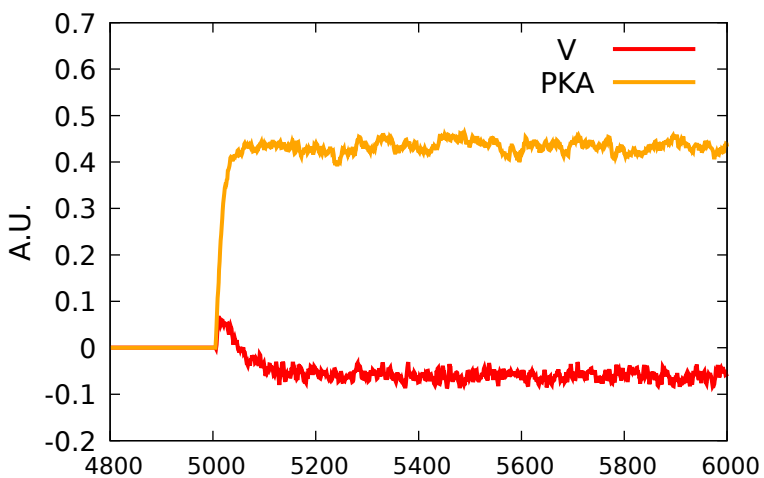NaC<sup>LOF</sup>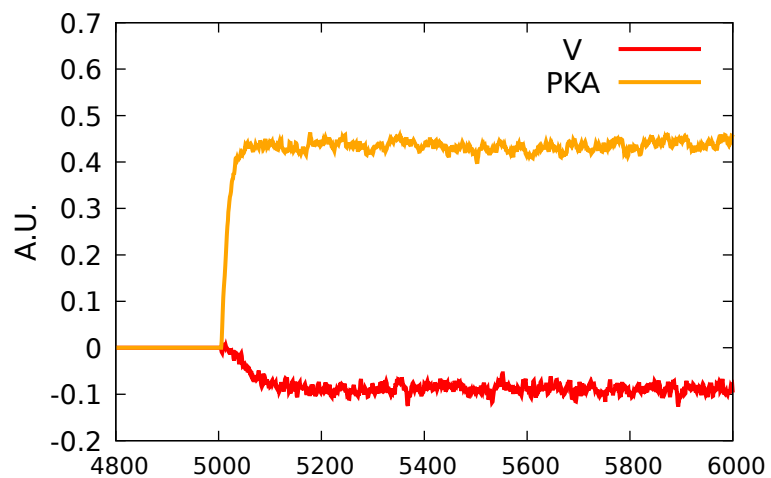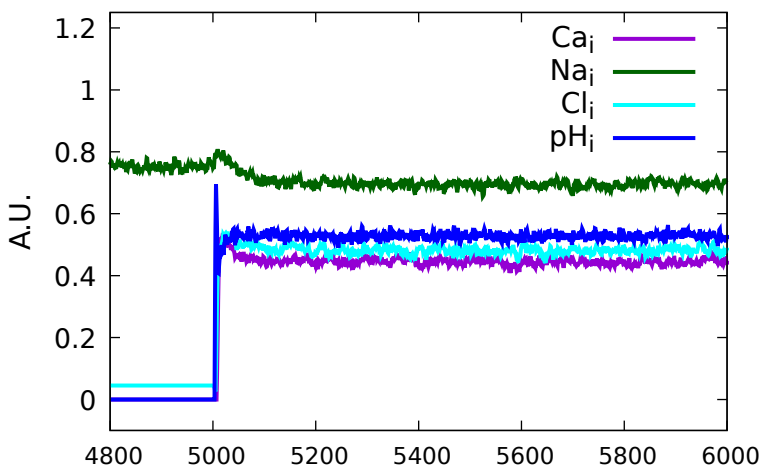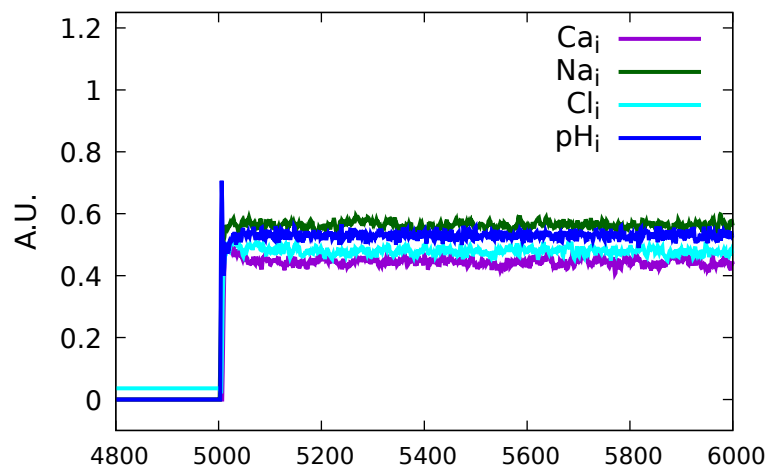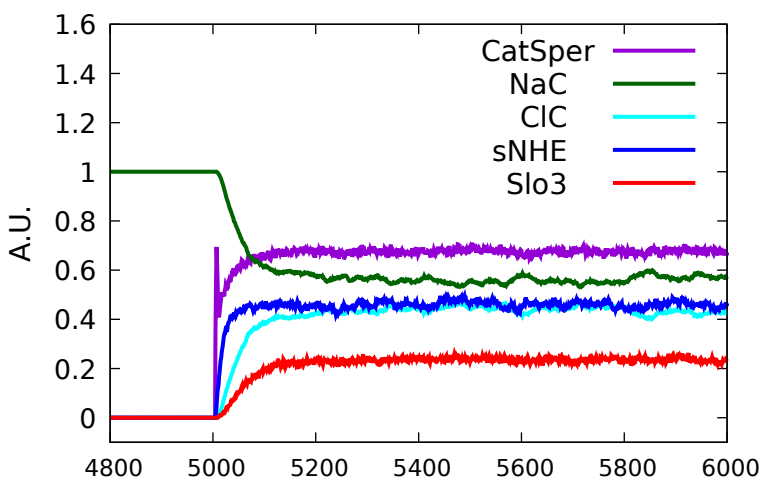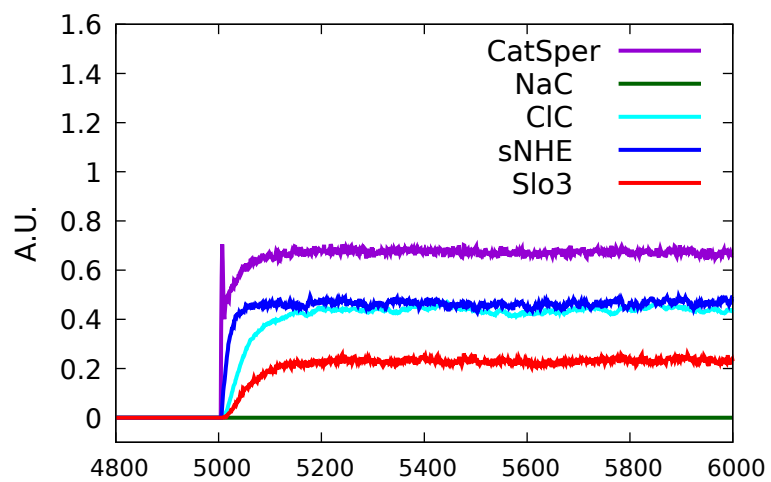

Supplement: S5 Fig — Averaged time series with variability, under an external stimulation of the addition of cholesterol acceptors and higher bicarbonate levels in the extracellular medium, of select set of variables under WT conditions (first column) and NaCLOF (second column). Membrane potential V hyperpolarizes, Nai decreases, Cai, Cli, pHi and PKA activity increase. Population size is 2 × 10^3^ sperm, the standard deviation used in introducing variability on ion transporter weights is D = 0.25. (PDF) [file pone.0245816.s006.pdf]

WT

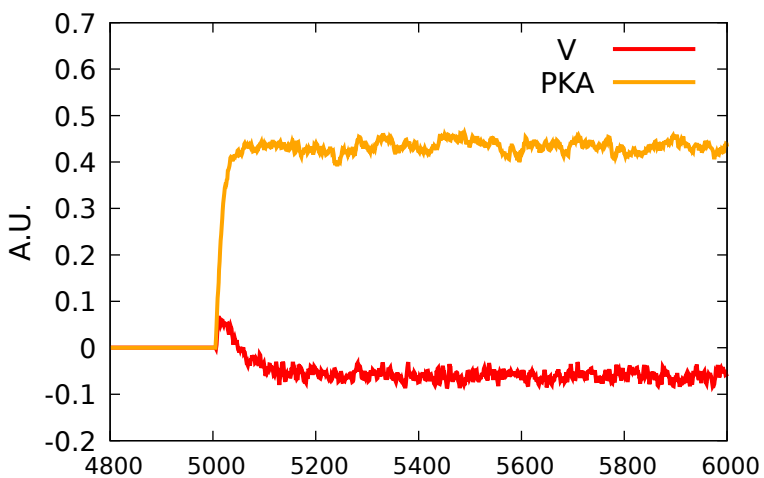Slo3<sup>LOF</sup>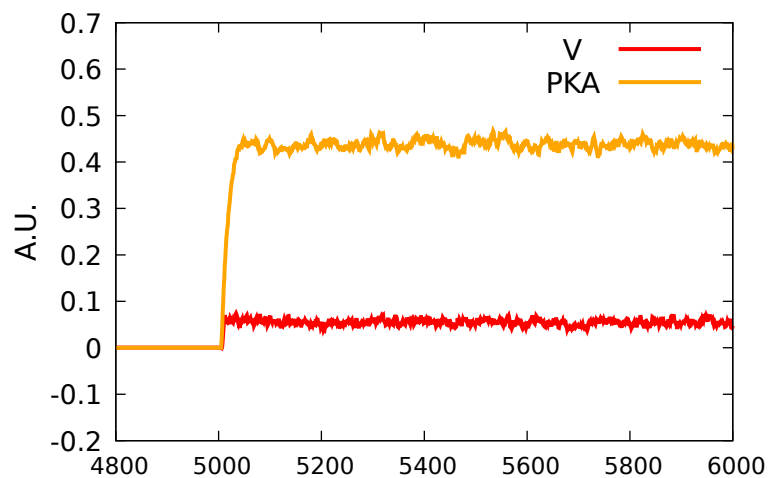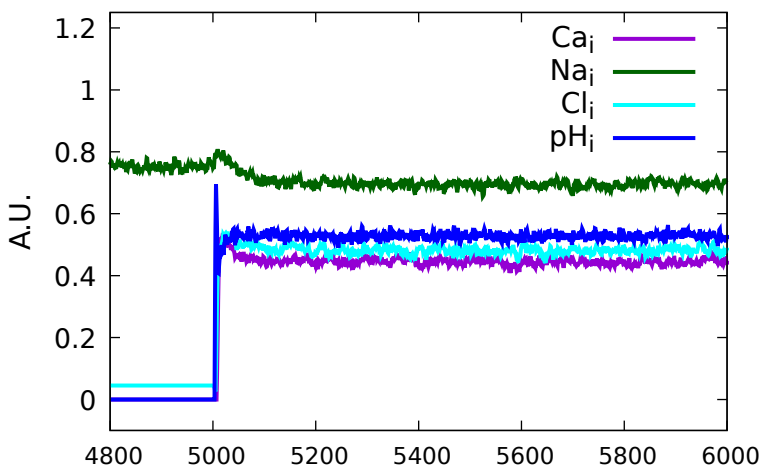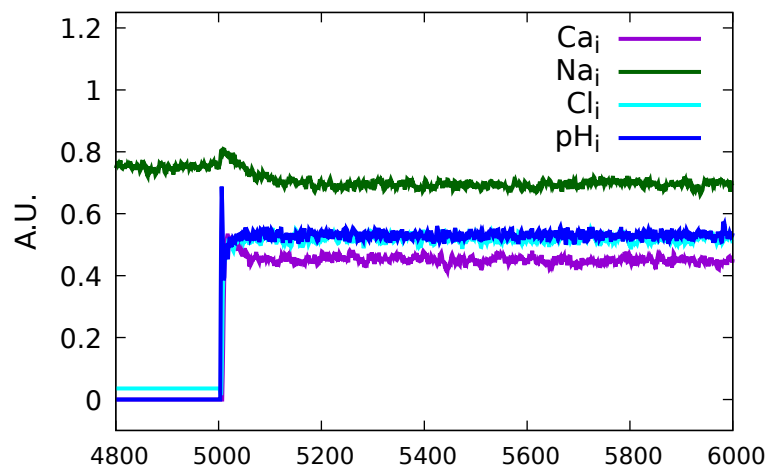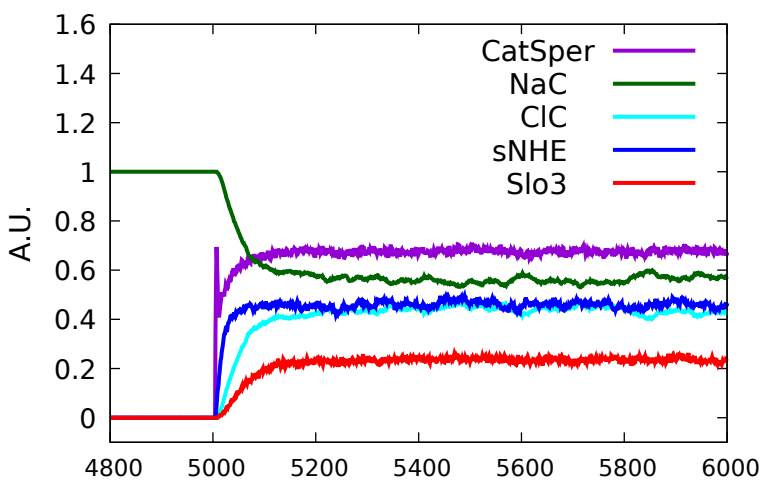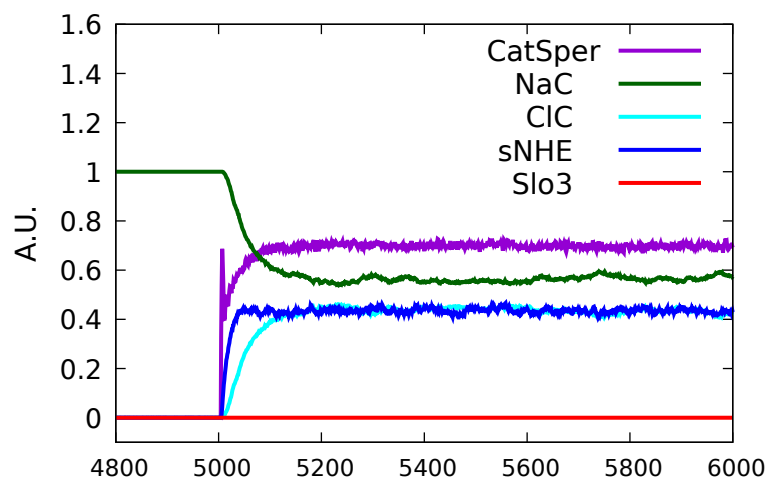

Supplement: S6 Fig — Averaged time series of select set of variables with variability, under an external stimulation of the addition of cholesterol acceptors and higher bicarbonate levels in the extracellular medium, under WT (first column) conditions and Slo3LOF (second column). Membrane potential V depolarizes, Nai decreases, Cai, Cli, pHi and PKA activity increases. Population size is 2 × 10^3^ sperm, the standard deviation used in introducing variability on ion transporter weights is D = 0.25. (PDF) [file pone.0245816.s007.pdf]

WT

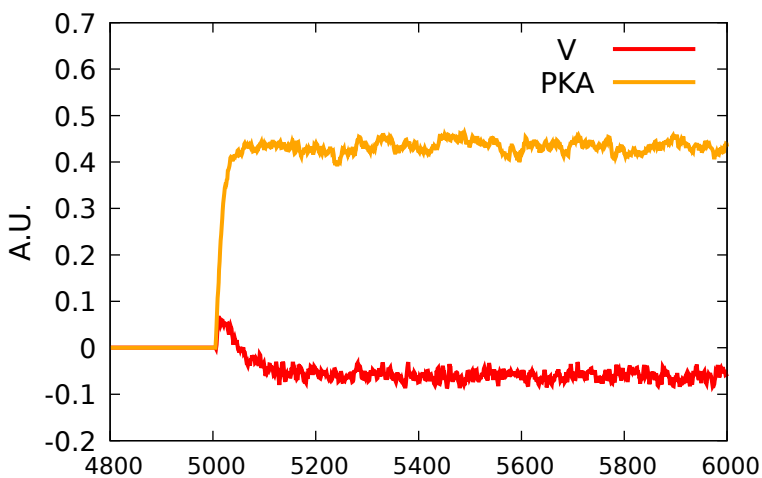PKA<sup>LOF</sup>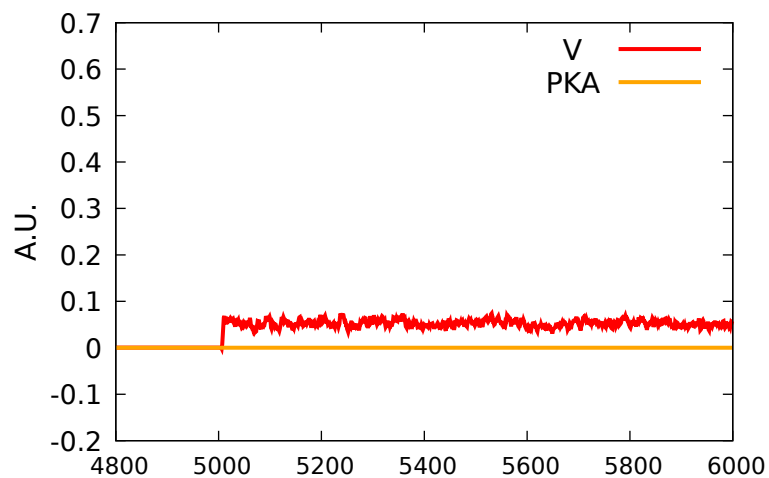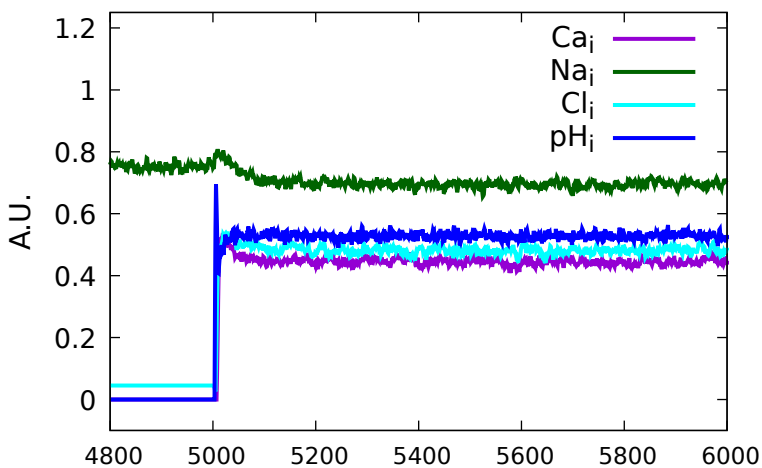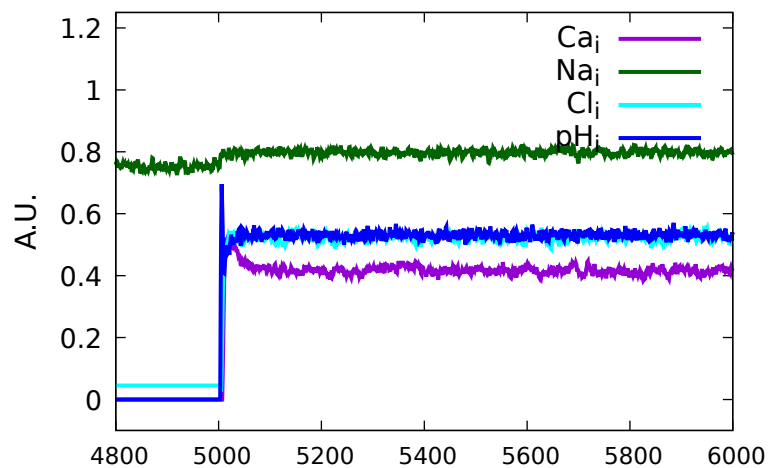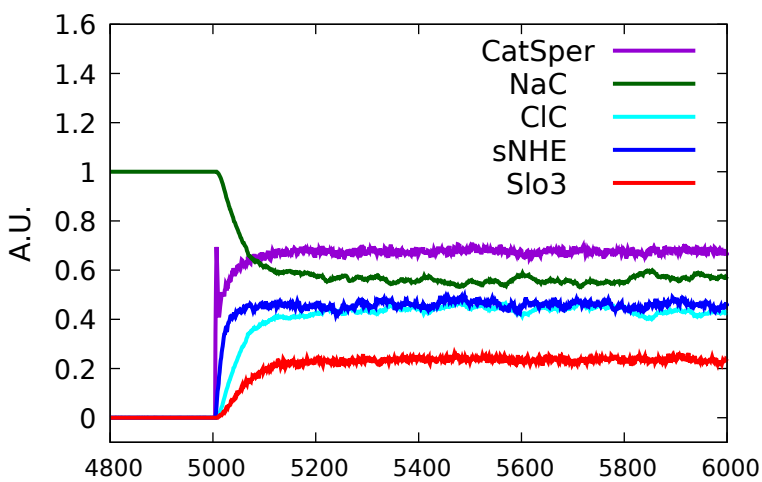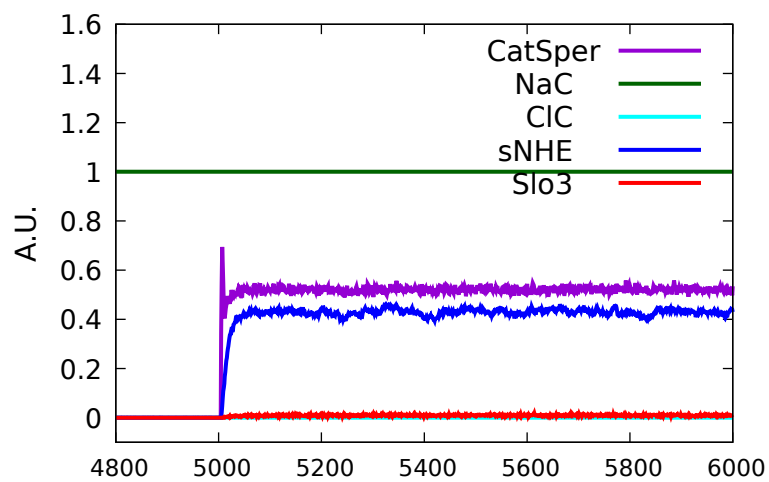

Supplement: S7 Fig — Averaged time series with variability, under an external stimulation of the addition of cholesterol acceptors and higher bicarbonate levels in the extracellular medium, of select set of variables under WT conditions (first column) and PKALOF (second column). Membrane potential V depolarizes, Nai, Cai, Cli and pHi increase, PKA activity goes to zero. Population size is 2 × 10^3^ sperm, the standard deviation used for introducing variability on ion transporter weights is D = 0.25. (PDF) [file pone.0245816.s008.pdf]

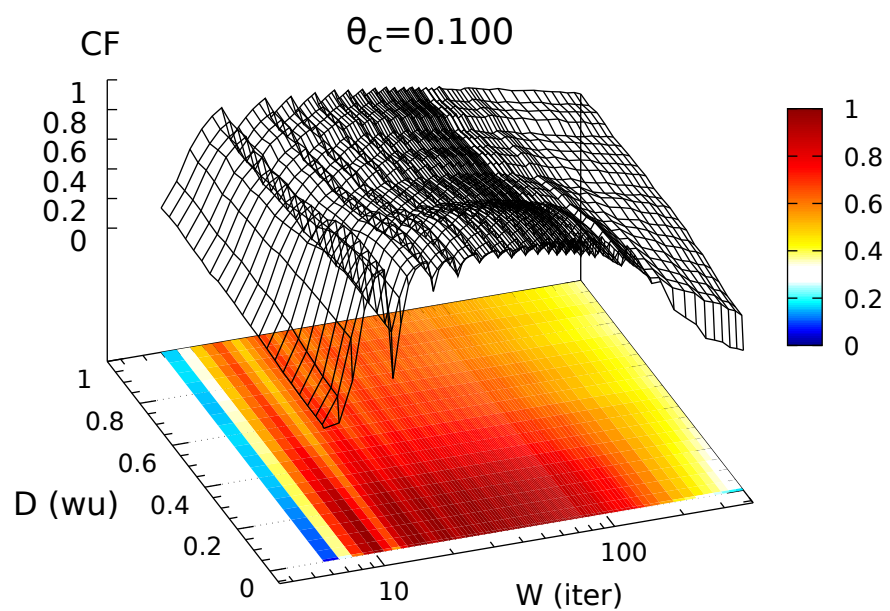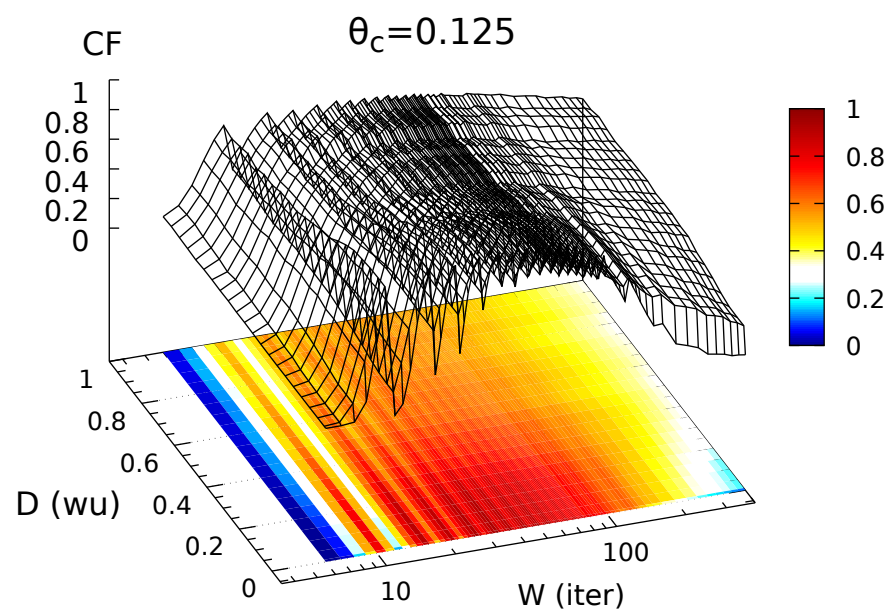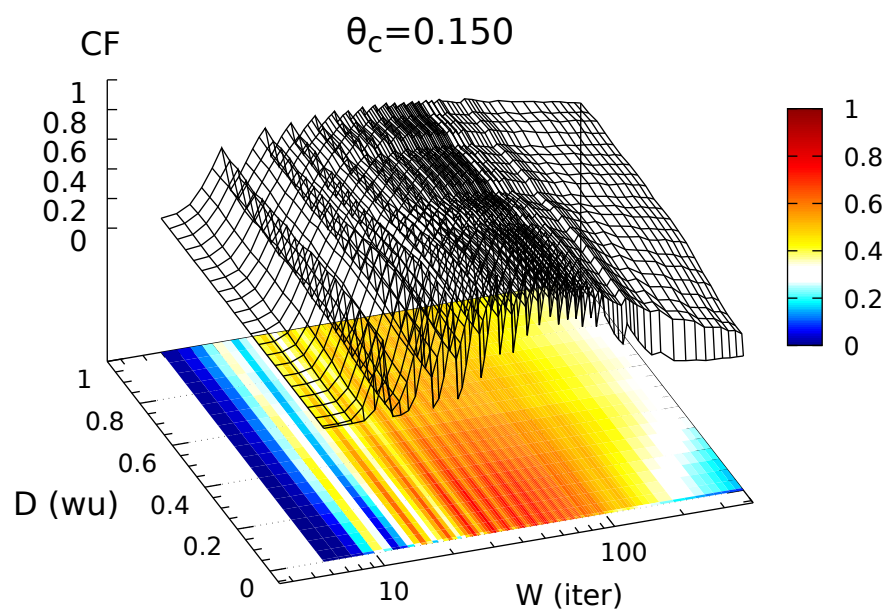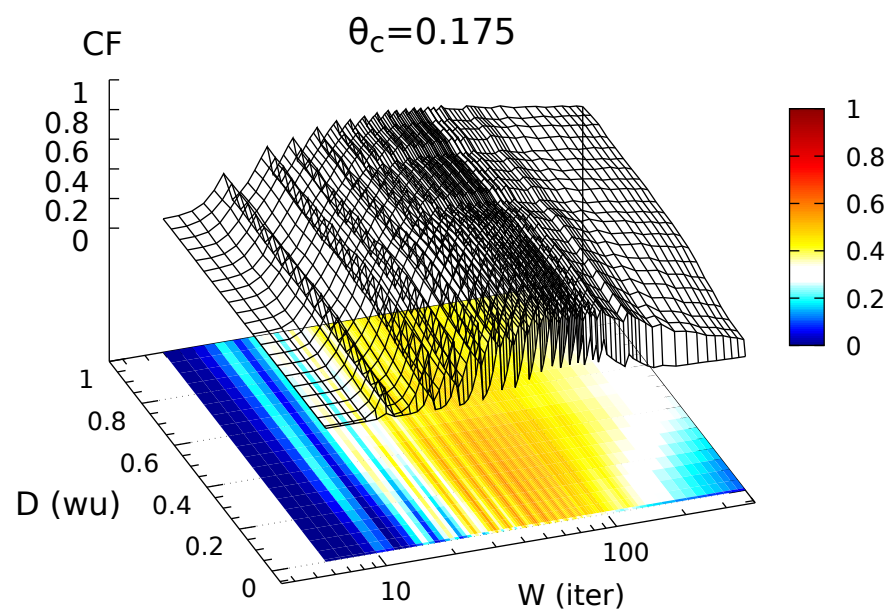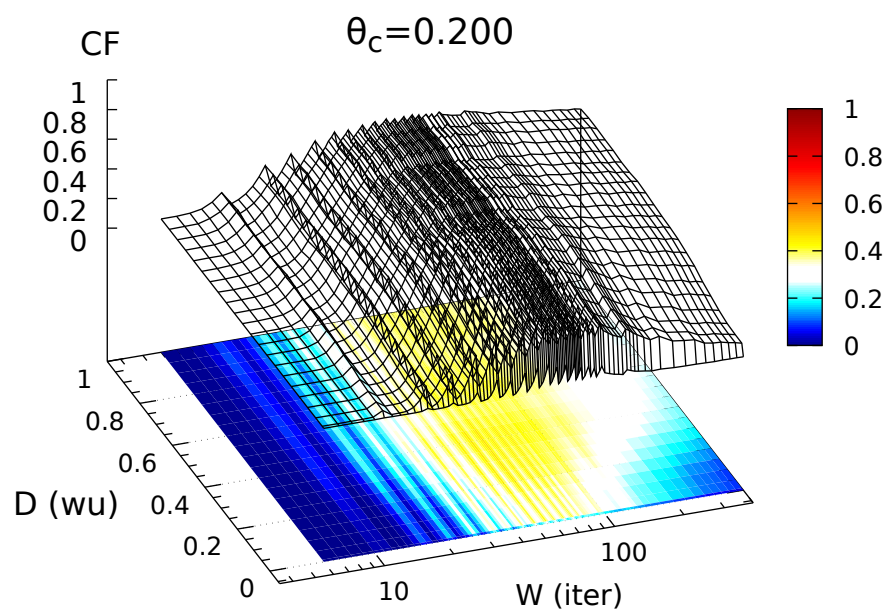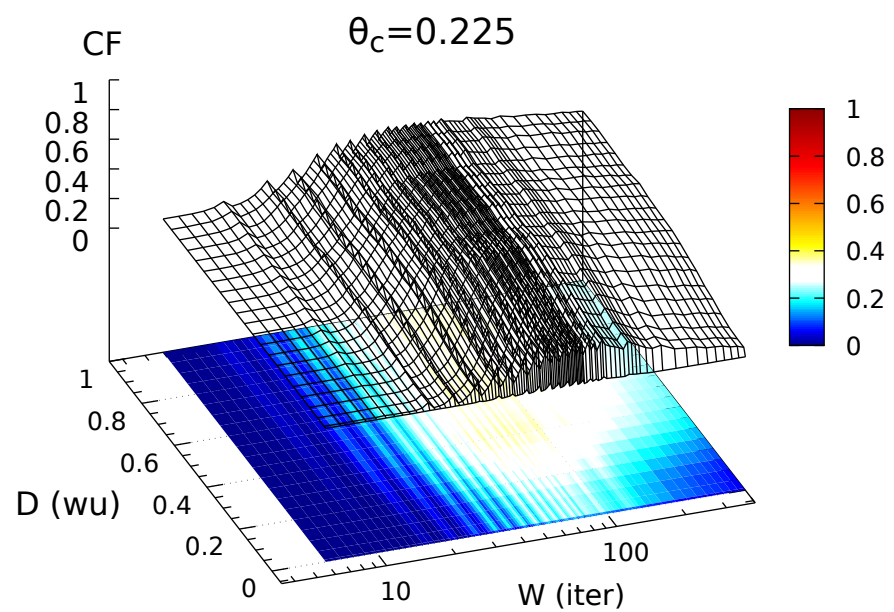

Supplement: S8 Fig — In all surfaces, each data point comes from 2 × 10^3^-sperm sized populations with 2 × 10^4^ iterations long simulations (5 × 10^3^ iterations at resting state and 1.5 × 10^4^ post stimulus iterations). The classification of capacitated vs. non-capacitated sperm is applied on the last 104 time iterations of each individual sperm. The color bar represents the level of capacitation fraction. Note that the white zone corresponds to a capacitation level of 30±5%, which is close to the levels typically observed for in vitro capacitation in wild-type sperm. (PDF) [file pone.0245816.s009.pdf]

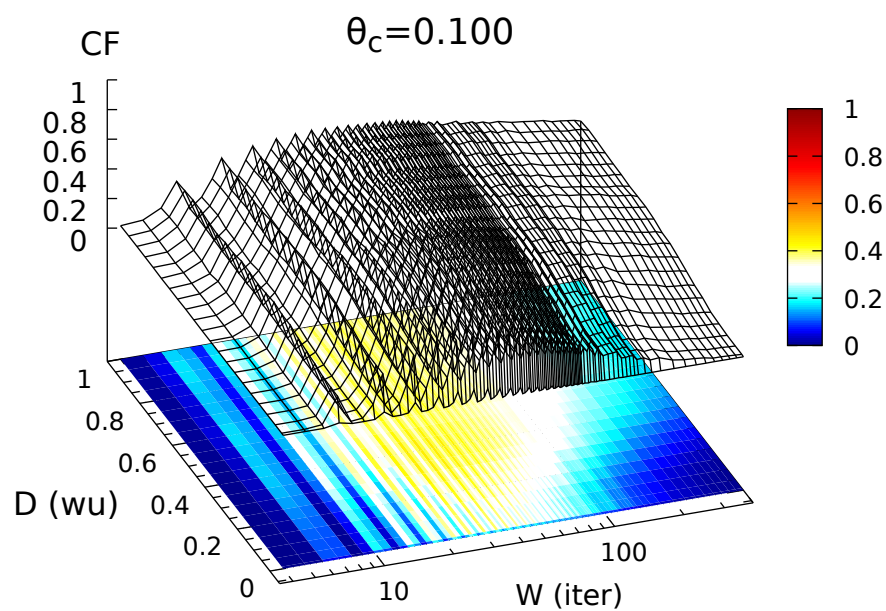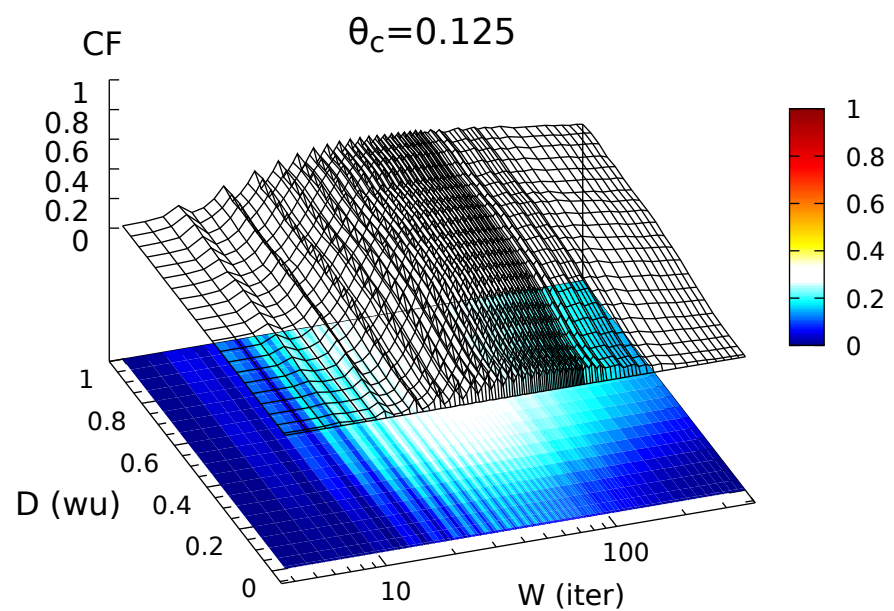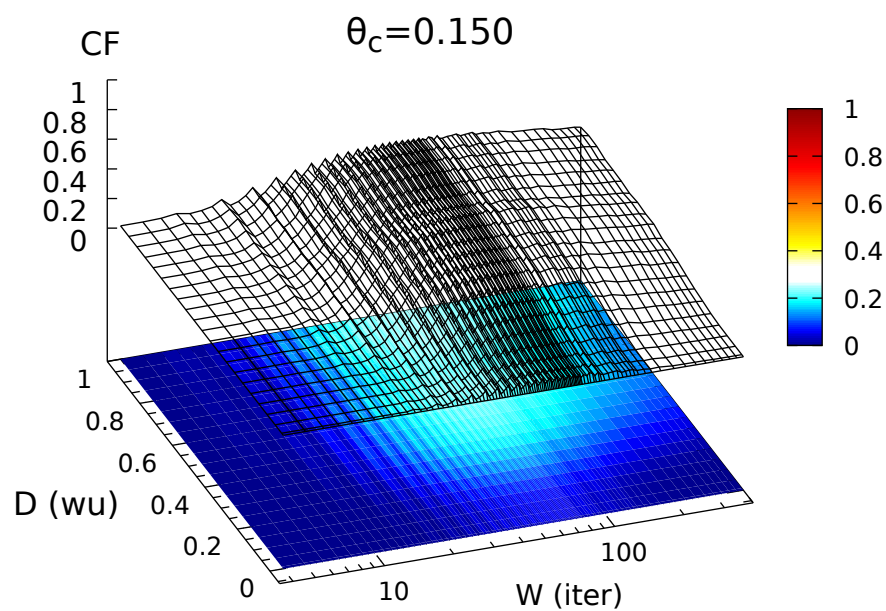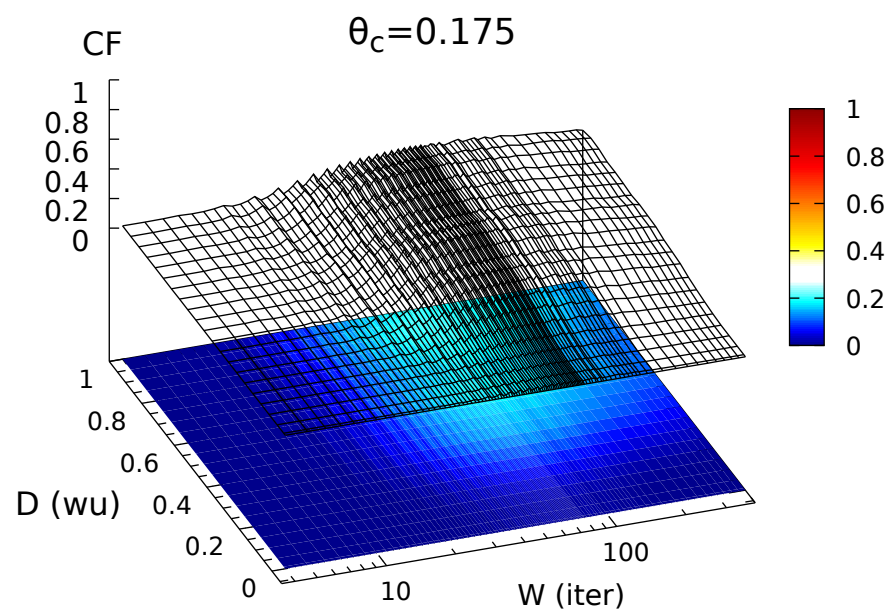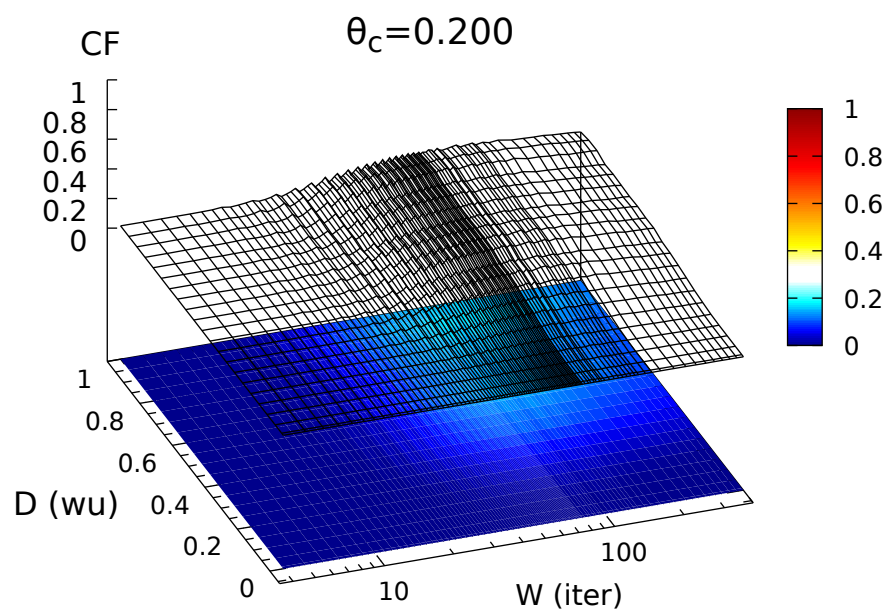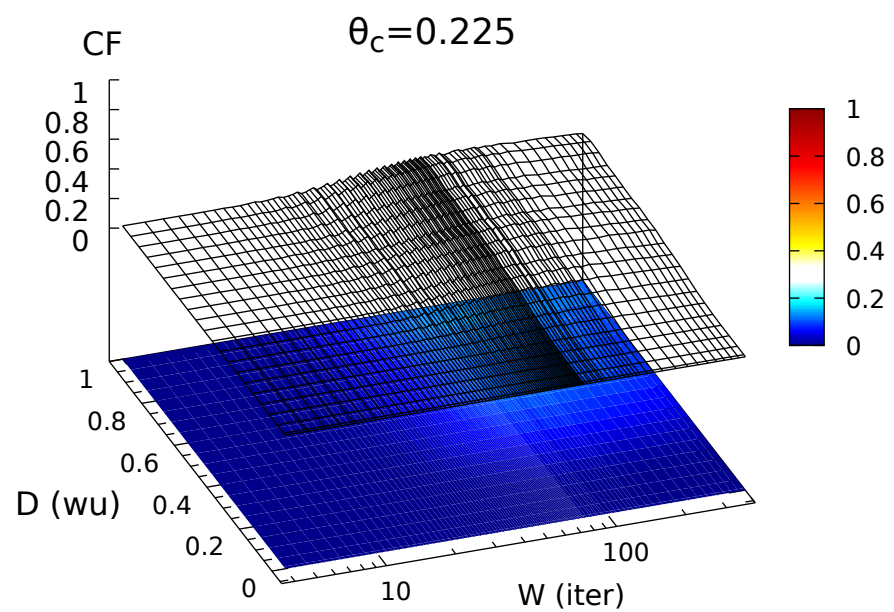

Supplement: S9 Fig — Surfaces are determined as in S8 Fig. (PDF) [file pone.0245816.s010.pdf]
